# Separation of Iron and Rare Earths from Low‐Intensity Magnetic Separation (LIMS) Tailings through Magnetization Roasting‐Magnetic Separation

**DOI:** 10.1002/open.202300059

**Published:** 2023-10-30

**Authors:** Shaochun Hou, Weiwei Wang, Bo Zhang, Wenjun Li, Chunlei Guo, Qiang Li, Erdou Li

**Affiliations:** ^1^ School of Chemical and Biological Engineering University of Science and Technology Beijing Beijing 100083 China; ^2^ State Key Laboratory of Bayan Obo Rare Earth Resource Researches and Comprehensive Utilization Baotou Research Institute of Rare Earths Baotou 014030 Inner Mongolia China

**Keywords:** LIMS tailings, magnetization roasting, magnetic separation: hematite, rare earth

## Abstract

Low‐intensity magnetic separation tailings (LIMS tailings) are a common by‐product obtained after magnetite magnetic separation. In this article, various techniques such as chemical analysis, X‐ray diffraction, ICP‐MS, and Mineral Liberation Analysis (MLA) were employed to investigate the LIMS tailings. The primary iron‐bearing mineral identified was hematite and rare earth minerals were monazite and bastnaesite. The main gangue species was fluorite with small amounts of dolomite and amphibole. Due to the weak magnetism of hematite and rare earth minerals, magnetic separation has low efficiency. However, magnetization roasting‐magnetic separation is an effective method to recover hematite. The present study focuses on the separation of iron and rare earth from LIMS tailings through magnetization roasting‐magnetic separation. The results demonstrate that with a roasting temperature of 650 °C, a roasting time of 60 min, a slurry concentration solid‐liquid ratio of 25 : 1, a rough magnetic field intensity of 0.16 T, and a selected magnetic field intensity of 0.10 T, the iron grade in the magnetic concentrate increases to 65.49 % and an iron recovery rate of 65.16 % can be achieved. The XRD patterns of magnetic separation concentrate show that the main mineral phases in concentrate are magnetite (Fe_3_O_4_) and fluorite (CaF_2_), which can be removed by grinding and reverse flotation fluorite to obtain a high‐grade iron concentrate. The REO grade of magnetic separation tailings is 11.98 %, and its recovery rate is 97.96 %. Consequently, rare earth can be effectively extracted and separated after the subsequent flotation‐leaching process.

## Graphical abstract

Low‐intensity magnetic separation (LIMS) tailings are a common by‐product obtained after magnetite magnetic separation, containing hematite and the rare earth minerals monazite and bastnaesite. Due to the weak magnetism of hematite and rare earth minerals, magnetic separation has low efficiency. The present study focuses on the separation of iron and rare earth from LIMS tailings through magnetization roasting‐magnetic separation. The results demonstrate that the iron grade in the magnetic concentrate can be increased to 65.49 % with an iron recovery rate of 65.16 %. Remaining fluorite (CaF_2_) can be removed by grinding and reverse flotation to obtain a high‐grade iron concentrate.

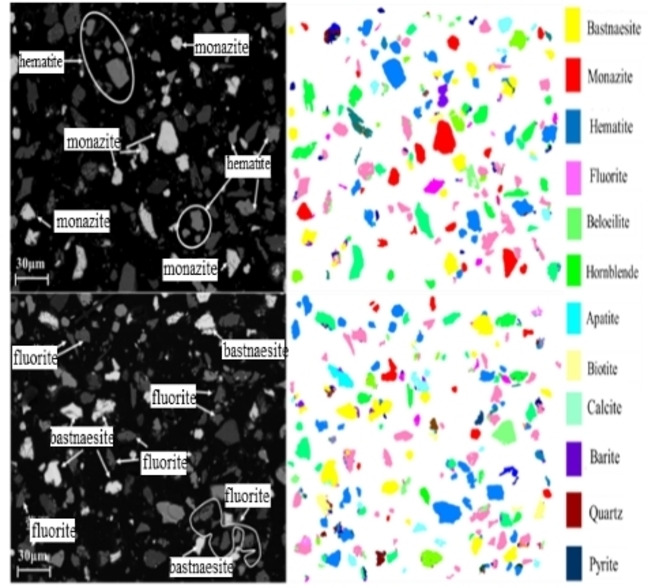

## Introduction

According to geological surveys, the Bayan Obo mine has been identified as the world‘s largest Fe‐REE‐Nb and polymetallic concomitant deposit.[^1^, ^2^] Since its development and utilization, the primary focus of exploiting the Bayan Obo mine has been based on low‐intensity magnetic separation incorporated with high‐intensity magnetic separation‐flotation.^[3]^ This approach focuses on the production of iron ore, with rare earth recovery being an auxiliary part.^[4]^ Currently, the utilization rate of rare earth resources is less than 20 %.[^5^, ^6^, ^7^] Consequently, a large portion of valuable elements is discharged into the tailings dam during the beneficiation process, resulting in an accumulation of 160 million tons of stacked tailings. This not only leads to resource wastage but also poses a significant environmental pollution risk.[^8^, ^9^, ^10^, ^11^, ^12^]

Aiming at resolving this problem, numerous investigations have been carried out to explore comprehensive utilization methods for stacked tailings. One traditional approach for iron extracting from tailings is through high‐intensity magnetic separation, which is typically conducted using pulsating high‐gradient magnetic separators. This approach can increase the iron grade from 15 % to more than 65 %.^[13]^ However, high‐intensity magnetic separation is not an appropriate scheme for the preconcentration of rare earth minerals due to their weak magnetic characteristics. In this regard, Qiu et al.^[14]^ studied the magnetic separation preconcentration technology of Yannuping rare earth ore, demonstrating that the grade of rare earth concentrate can be enhanced from 1.52 % to 7.40 %. Consequently, it is a challenge to separate hematite and rare earth minerals using high‐intensity magnetic separation due to their similar weak magnetic properties.

In order to resolve this problem, reduction roasting‐magnetic separation technology was proposed for extracting metals from refractory iron‐containing resources such as red mud,[^15^, ^16^] copper slag,^[17]^ iron tailings,^[18]^ vanadium tailings,^[19]^ and cyanide tailings.^[20]^ Furthermore, the magnetization roasting method is widely employed to convert weakly magnetic hematite and rare earth minerals into magnetite, thereby enhancing their magnetic properties and improving the separation efficiency. However, there are limited comprehensive investigations on the separation of iron and rare earth using this technique. In this study, magnetization roasting‐magnetic separation is employed to separate iron and rare earth from low‐intensity magnetic separation tailings (LIMS tailing). The research focuses on investigating the impact of affecting parameters such as roasting temperature, magnetic field strength, slurry flow rate, and slurry concentration on the separation efficiency of iron and rare earth. This article provides a basis for extracting rare earth and iron from stacked tailings.

## Materials and Methods

### Materials

**Figure 1:**
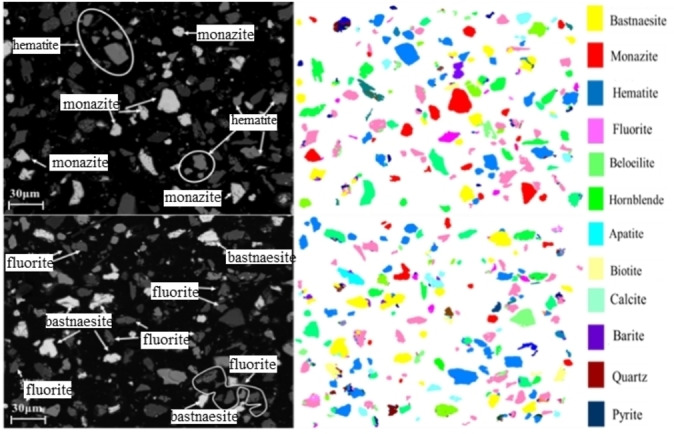
MLA images of the LIMS tailings.

**Figure 2:**
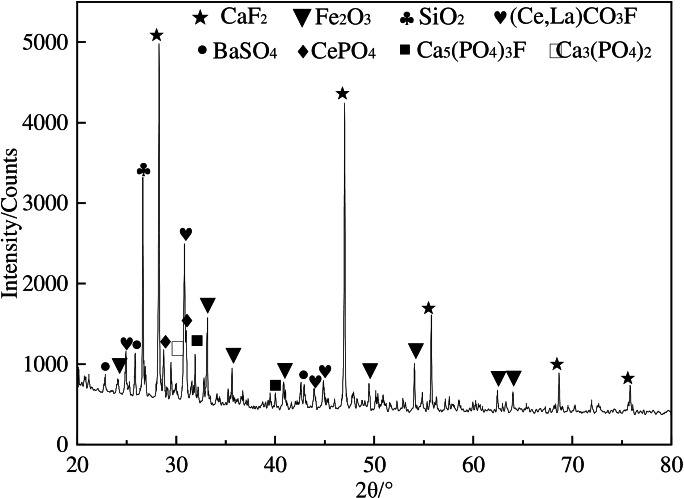
XRD patterns of the LIMS tailings

**Figure 3:**
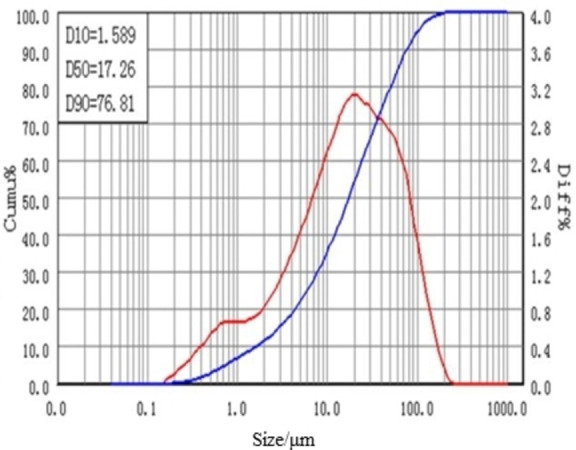
Particle size distribution of the LIMS tailings

The present research utilizes the LIMS tailings obtained from a mineral processing company located in Bayan Obo, Inner Mongolia, China as the research material. Table 1 presents the main chemical composition of the research material, indicating that the tailings primarily consist of TFe (14.38 wt %), REO (9.60 wt %), Sc_2_O_3_ (0.012 wt %), CaO (22.99 wt %), F(12.57 wt %), SiO_2_(11.43 wt %). It should be indicated that TFe, REO and Sc_2_O_3_are the main recycling elements. The REO are mainly composed of La_2_O_3_, CeO_2_, Pr_6_O_11_, and Nd_2_O_3_, accounting for 97.9 % percent of the tREO. Figure 1 and Table 2 provide the MLA analysis results. Meanwhile, the XRD analysis results are shown in Figure 2. Furthermore, Figure 3 illustrates the particle size distribution of the LIMS tailings. The mineral composition results and particle size distribution reveal that the LIMS tailings have complex ore phases, mainly including hematite, fluorite, bastnaesite, amphibole, and dolomite phases. It is also observed that the mineral particles are very fine, and almost 79.78 % of particles are smaller than 50 μm. The hematite, bastnaesite, and monazite phases in LIMS tailings are in the form of monomers or fine inclusions within other minerals, which presents challenges for the extraction process.

**Table 1:** Chemical composition of the LIMS tailings ([wt %]).

CaO	TFe	F	SiO_2_	REO	BaO	MgO	S	Na_2_O	FeO	Nb_2_O_5_	Sc_2_O_3_	ThO_2_
22.99	14.38	12.57	11.43	9.60	4.10	2.50	1.76	0.95	1.51	0.17	0.012	0.056
K_2_O	MnO	TiO_2_	P_2_O_5_	Al_2_O_3_	Y_2_O_3_	La_2_O_3_	CeO_2_	Pr_6_O_11_	Nd_2_O_3_	Sm_2_O_3_	Eu_2_O_3_	Gd_2_O_3_
0.35	1.09	0.90	3.53	1.12	0.04	2.47	5.01	0.47	1.45	0.10	0.017	0.045

**Table 2:** Composition and main components of the LIMS tailings.

Mineral	Content [wt %]	Mineral	Content [wt %]
Magnetite (Fe_3_O_4_)	1.15	Bastnasite (CeCO_3_F)	10.5
Hematite (Fe_2_O_3_)	15.36	Monazite (CePO_4_)	2.74
Pyrite (FeS_2_)	0.76	Apatite (Ca_5_(PO4)_3_)	6.47
Pyrrhotite (Fe_1‐x_ S)	0.31	Fluorite (CaF_2_)	25.17
Dolomite (CaMg(CO_3_)_2_	6.42	Barite (BaSO_4_)	5.51
Quartz(SiO_2_)	1.01	Amphibole (Ca_2_Mg_5_Si_8_O_22_(OH)_2_	17.29
Feldspar (KAlSi_3_O_8_)	0.43	Mica (KAl_2_(AlSi_3_O_10_)(OH)_2_	5.75

The chemically pure sodium hydroxide and calcium hydroxide were used as the annexing agent while coal was utilized as the reductant material. The coal composition analysis is presented in Table 3.

**Table 3:** Analysis of coal composition ([wt %]).

Moisture	Ash	Volatile component	Fixed carbon	O
6.3	6.8	7.22	79.68	2.35

### Experimental and analytical methods

Prior to tests, the LIMS tailings were mixed with calcium hydroxide, sodium hydroxide, and coal in a mass ratio of 1 : 0 : 04 : 02 : 0.02. A total of 400 g of this mixture was placed into a graphite crucible. The crucible was then placed into a muffle furnace (SX2‐4‐10 Shanghai Kexiao Scientific Instrument Co., LTD, China) that had been preheated to the desired roasting temperature. After the specified reaction time was elapsed, the graphite crucible was removed from the furnace. To rapidly cool the samples, they were immediately transferred to ice water. once the roasted samples were cooled down to room temperature, they were taken out for magnetic separation testing.

The LIMS tailings and experimental products were dried at 100 °C for 60 minutes and then ground to 0.074 mm in a grinder. Subsequently, an X‐Ray Fluorescence (XRF) and an Inductively Coupled Plasma Mass Spectroscopy (ICP‐MS) were employed for chemical analysis. The samples were dissolved with sodium hydroxide and sodium peroxide at 750 °C, extracted with hydrochloric acid, and then determined by ICP‐MS. XRD analysis was performed on LIMS tailings and roasted samples using a Smartlab diffractometer with Cu Kα radiation, covering a 2θ range of 10° to 100°. The data obtained from these analyses were processed and analyzed using Jade and Origin software. To perform mineralogical analyses, a Mineral Liberation Analyzer (MLA) was utilized in the experiments. The analyzer primarily consisted of a standard SEM (FEI Quanta 600, Germany) and an energy‐dispersive X‐ray analyzer (EDAX; Genesis, Germany), and a powerful software package.

## Results and Discussions

### Roasting temperature

**Figure 4:**
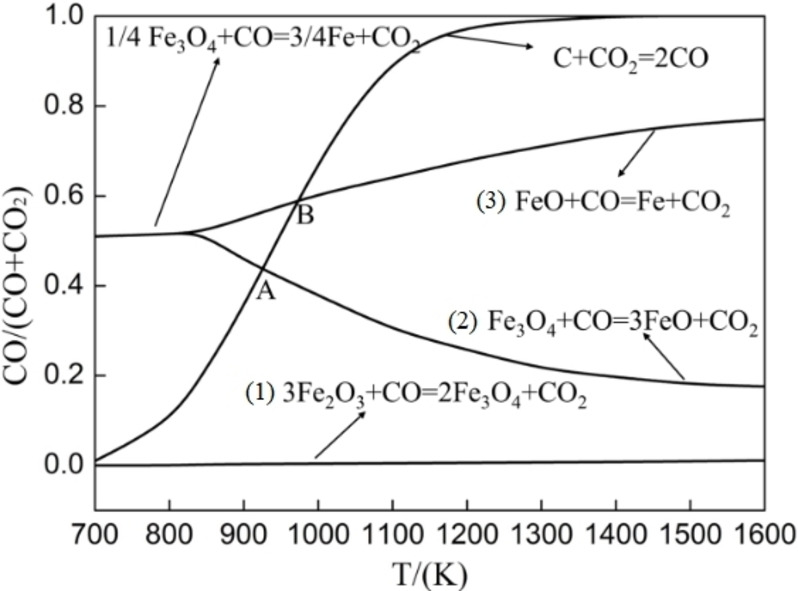
Gas‐phase equilibrium diagram of iron oxide reduced by solid carbon.

Figure 4 illustrates the equilibrium diagram of iron oxide reduction under standard conditions. It is observed that the curve of reaction (1) approaches the horizontal axis, indicating that the trace amount of CO can effectively reduce hematite to magnetite, and this reaction is very prone to occur. The carbon gasification reaction curve in reactions (2) and (3) exhibits two intersection points labeled A (at 950 K) and B (at 990 K). When the temperature is less than 950 K, the equilibrium CO concentration of the system determines that the reduction product is Fe_3_O_4_. However, when the temperature ranges from 950 to 990 K, the predominant reduction product shifts to FeO. When the temperature exceeds 990 K, the main reduction product is Fe. It is worth noting that FeO has detrimental effects in the one‐step roasting experiment and should be avoided. Consequently, the roasting temperature should be maintained below 950 K (677 °C).

To determine the optimal roasting temperature, the roasting time was set to 60 minutes. Moreover, iron content and magnetic iron content in raw materials were measured before and after roasting. Reduction magnetic susceptibility^[21]^ is a widely used measure to evaluate the moderate reduction effect of ore samples. The magnetisability index is defined as follows [(Eq. 1]:

(1) $$magnetisability=\frac{Total\;iron\;content\;in\;samples}{Ferrous\;iron\;content\;in\;samples}=\frac{\omega TFe}{\omega FeO}$$

**Figure 5:**
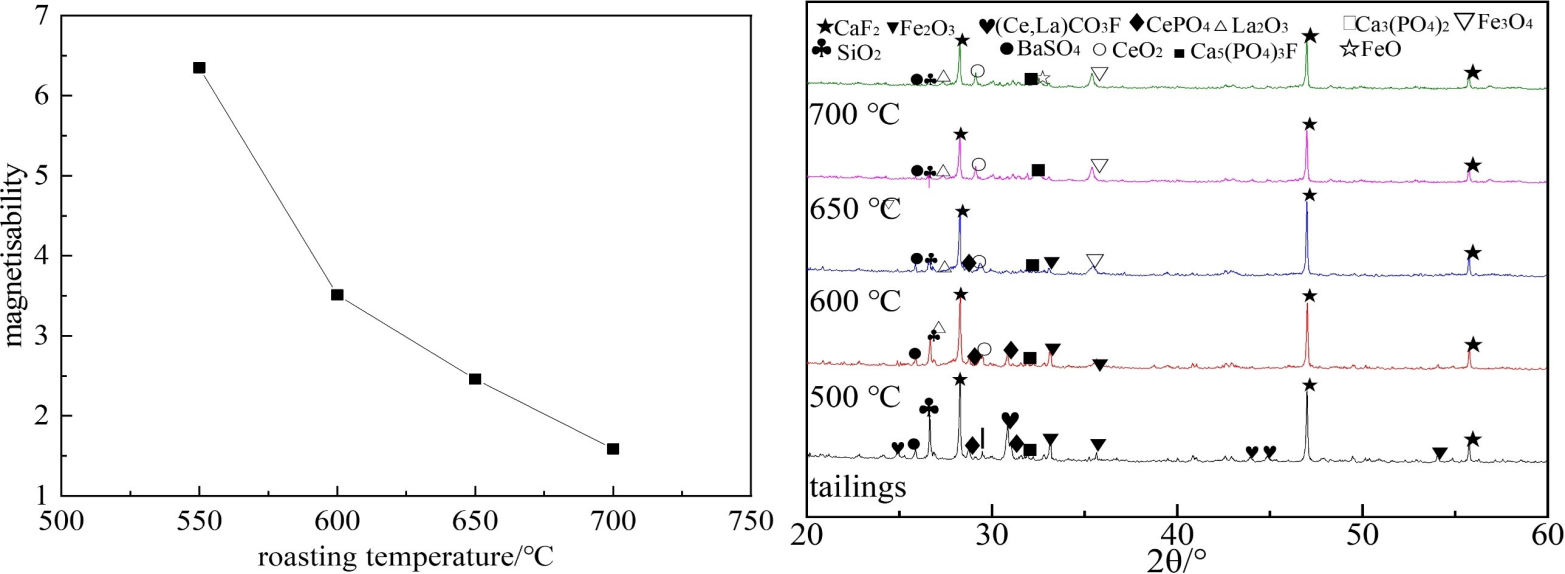
Influence of roasting temperatures on the magnetizability index.

The ideal magnetisability index, which corresponds to the theoretical grades of hematite and magnetite, is 2.33. When this index exceeds 2.33, the concentration of Fe^2+^ ions in the product is low, while the concentration of Fe^3+^ ions is high, and the reduction is incomplete. On the contrary, excessive reduction occurs. Figure 5 shows that when the temperature ranges from 550 °C to 700 °C, the magnetisability has a positive correlation with the roasting temperature. For a roasting temperature of 650 °C, the magnetisability is 2.46, which aligns with the theoretical value of 2.33. This suggests that 650 °C is the appropriate magnetization roasting temperature, and the optimal conversion of Fe_2_O_3_ to Fe_3_O_4_ can be achieved at this temperature. Similar results can be achieved from XRD analysis. Therefore, 650 °C is selected as the roasting temperature for the test.

### Roasting time

**Figure 6:**
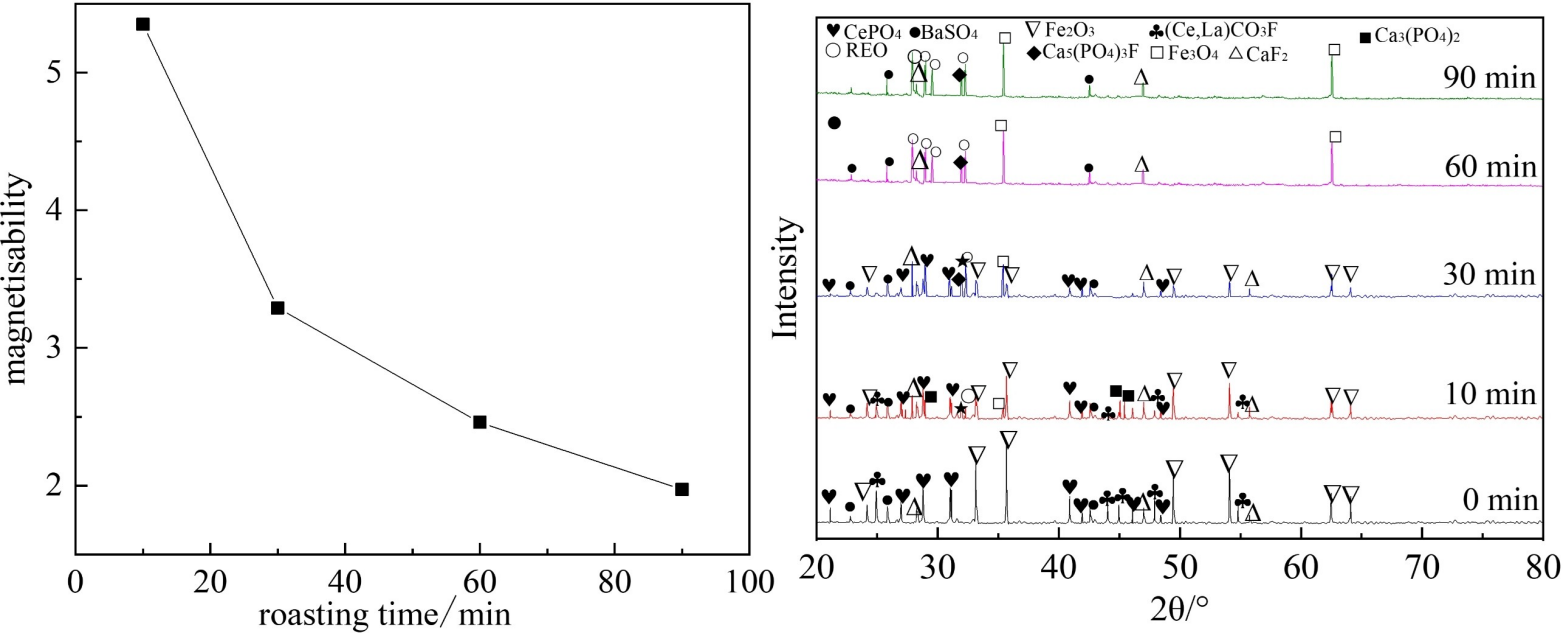
The impact of roasting time on the magnetizability.

**Figure 7:**
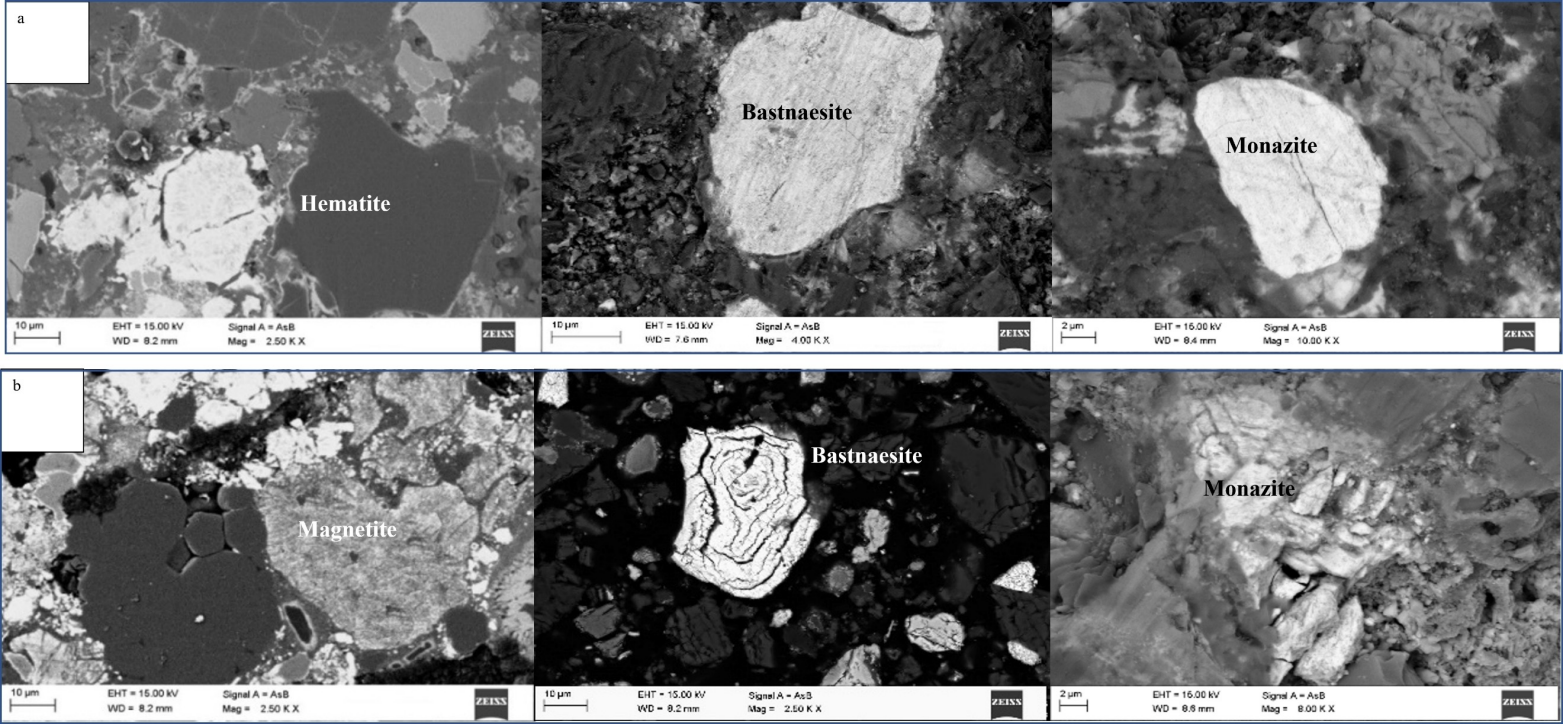
SEM images of roasted samples at 650 °C for different roasting times: (a) 0 min, (b) 60 min.

Studies show that roasting time is a crucial factor in reducing hematite to magnetite.^[22]^ An insufficient roasting time results in an incomplete chemical reaction. However, a too‐long roasting leads to excessive reduction and waste of resources. The raw material used in this experiment is a mixture of tailings and reducing agents. To optimize the roasting time, experiments were conducted with different roasting times, including 10 min, 30 min, 60 min, and 90 min, and the obtained results are presented in Figure 6. It is observed that as the roasting time increases, the magnetisability decreases gradually and iron reduces. At a roasting time of 60 minutes, the corresponding magnetisability reaches 2.46, which is consistent with the theoretical value. However, the magnetisability drops to 1.97 at a roasting time of 90 minutes, indicating that over‐reduction occurs and Fe_3_O_4_ reduces to FeO. It should be indicated that a too‐long roasting time not only increases energy consumption but also reduces strong magnetic Fe_3_O_4_ to FeO, which is not conducive to the subsequent magnetic separation. Figure 7 illustrates SEM images of roasted samples at 650 °C for 0 min and 60 min. It is observed that the weakly magnetic hematite reduces to strongly magnetic magnetite after roasting, facilitating the separation of rare earth minerals and magnetite from stacked tailings. Meanwhile, the structure of bastnaesite and monazite becomes loose and porous, which is beneficial for the subsequent smelting and separation processes. Consequently, the optimal roasting time is determined to be 60 minutes.

### Slurry concentration

**Figure 8:**
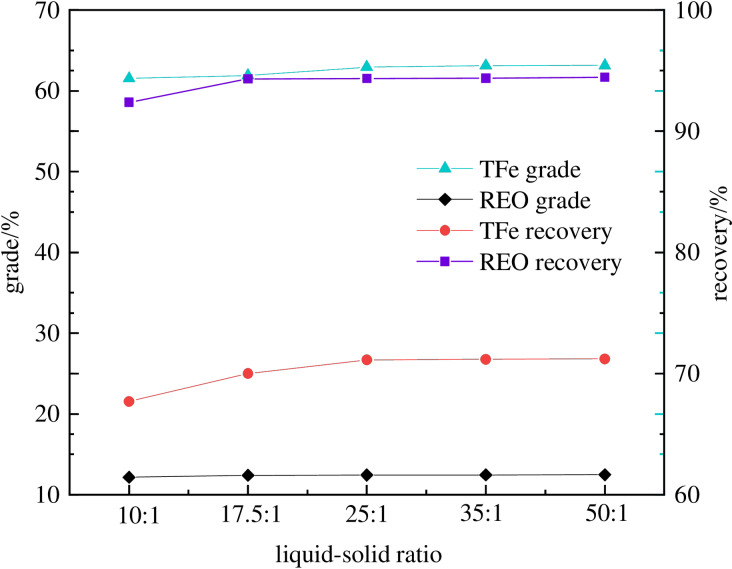
The impact of slurry concentration on the separation efficiency of TFe‐REO.

To analyze the effects of the slurry concentration on the magnetic separation, tests were conducted under a magnetic field strength of 0.14 T. Figure 8 reveals that as the slurry concentration increases, the recovery of iron and rare earth increases. This may be attributed to the good wettability of mineral particles at high slurry concentrations, which results in the dispersion of mineral particles in the slurry. On the contrary, when the slurry concentration is low, the viscosity of the slurry is high, the number of mineral particles in the unit volume slurry is large, the interaction between mineral particles is large, and the mineral particles are not fully dispersed. Under this condition, gangue forms in which rare earth minerals are mixed with magnetic iron. This phenomenon reduces the iron grade of magnetic separation concentrate. When the liquid‐to‐solid ratio is 25 : 1, the iron recovery of the concentrate is 69.13 % and the iron grade is 62.94 %, the REO recovery of tailings is 94.36 % and the REO grade is 12.43 %. The results demonstrate that as the liquid‐to‐solid ratio increases, the iron and REO recovery do not increase significantly. This is because the mineral particles are already fully wet‐dispersed so further increase in the liquid‐to‐solid ratio has a negligible improvement impact. Considering the grade and yield, the optimal liquid‐solid ratio was determined to be 25 : 1.

### Roughing magnetic intensity

**Figure 9:**
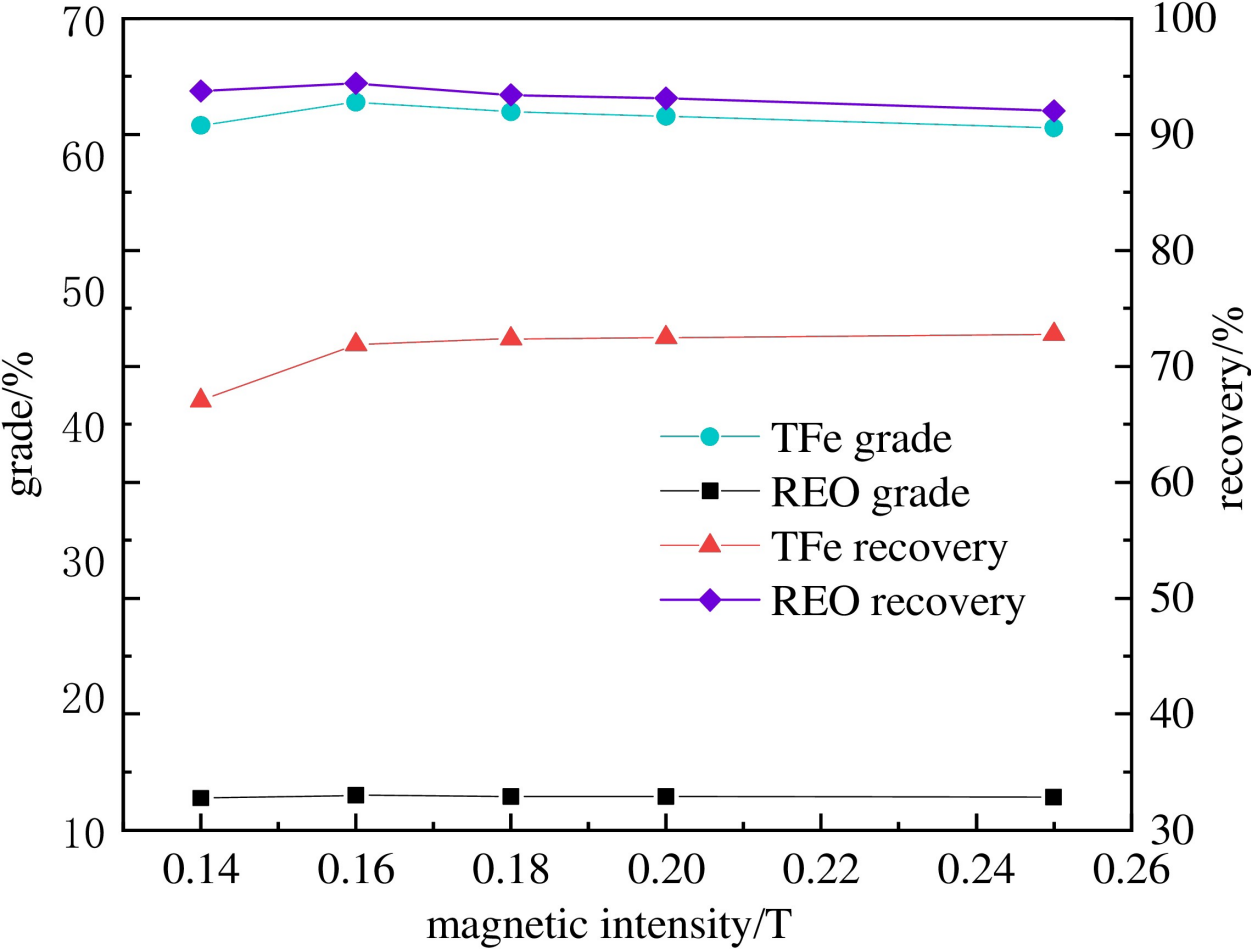
Influence of roughing magnetic intensity on the separation of TFe‐REO.

Based on findings in previous sections, samples with optimal parameters were utilized in the magnetic separation tests. Tests were conducted at a roasting temperature of 650 °C, a roasting time of 60 minutes, a slurry flow rate of 0.5 cm/s, a liquid‐to‐solid ratio of 25 : 1, and various magnetic field intensities including 0.14 T, 0.16 T, 0.18 T, 0.20 T, and 0.25 T. Figure 9 shows that as the roughing magnetic field strength increases from 0.14 T to 0.16 T, the recovery rate and grade of iron and REO increase. It is observed that as the magnetic field intensity increases, iron recovery increases while iron grade decreases, and both the grade and recovery rate of REO decrease. Due to the agglomeration of magnetic and fine particles, which leads to many weak magnetic bonds between minerals and gangue, miscellaneous minerals may also enter into the crude concentrate. Moreover, a part of REO is engrained so the grade of iron in the crude concentrate decreases, and the grade of REO in the crude tailings declines. Under a magnetic field intensity of 0.16 T, the iron recovery is 68.86 %, the REO recovery is 94.39 %, the iron grade is 63.81 %, and the REO grade is 12.56 %. As the magnetic field intensity increases, the iron recovery does not increase significantly, the iron grade decreases, and the REO yield grade decrease too. Accordingly, the optimal magnetic field intensity in this experiment is 0.16 T.

### Magnetic intensity

**Figure 10:**
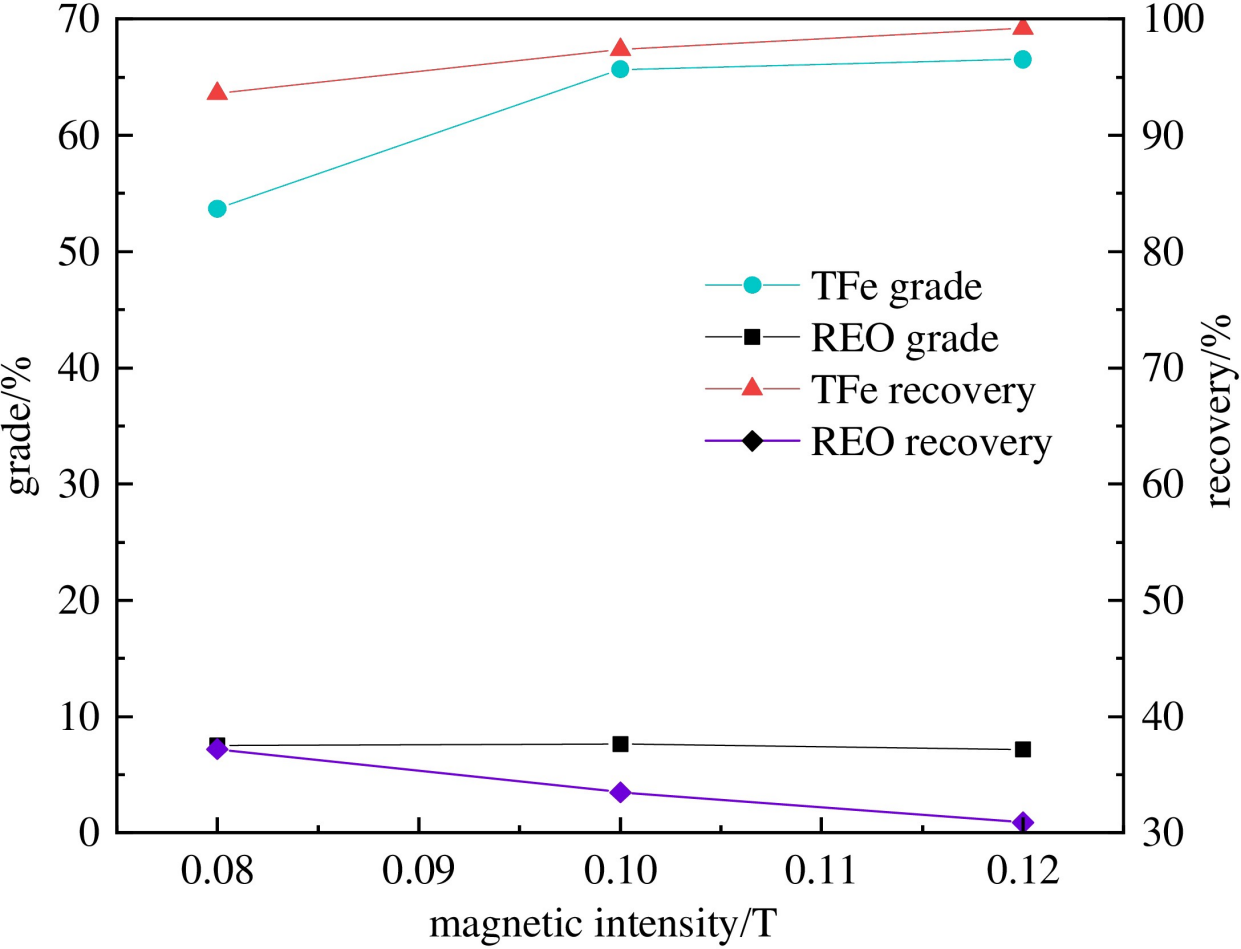
Influence of concentration magnetic intensity on the separation of TFe‐REO.

The coarse concentrate obtained under a magnetic field intensity of 0.16 T was ground to dissociate part of the interlocked minerals. The magnetic field intensity of the selection was 0.08 T, 0.10 T, and 0.12 T, and the slurry concentration was liquid with a solid ratio of 25 : 1. Figure 10 reveals that as the magnetic field strength increases, the grade and recovery of iron increase, while the recovery of REO gradually decreases. The REO grade reaches its highest value at 0.10 T. This is because as the magnetic field strength increases, the imposed magnetic force on magnetic mineral particles increases, and some weak magnetic particles associated with gangue are also recovered. Considering the recovery and grade of iron and REO, a magnetic intensity of 0.10 T is selected as the optimal value.

### Comprehensive condition experiment

In the foregoing sections, the single factors were analyzed, and optimized values were obtained independent of other parameters. More specifically, the optimal separation of LIMS tailings was obtained with a roasting temperature of 650 °C, a roasting time of 60 min, a roughing magnetic field intensity of 0.16 T, a concentration magnetic field intensity of 0.10 T, and a slurry concentration solid‐liquid ratio of 25 : 1. The experimental results at optimized conditions are shown in Table 4. It is observed that the iron grade of the obtained iron concentrate is 65.16 %, and the recovery is 65.49 %. In the literature, the high intensity magnetic separation‐flotation method is used to recover the iron in the LIMS tailings, and the grade and recovery are only 64.45 % and 58.47 %. Compared with this process, the iron concentrate (the grade is greater than 65 %)obtained by the magnetization roasting‐magnetic separation method can be directly used as the raw material for blast furnace iron making after subsequent ore blending treatment.

**Table 4:** Experimental results of comprehensive conditions.

Sample	Yield [%]	TFe		REO	
		Recovery [%]	Grade [%]	Recovery [%]	Grade [%]
Magnetic concentrate	13.98	65.49	65.16	2.04	2.71
Magnetic tailing	86.02	34.51	5.58	97.96	11.98
Roasted ore	100	100	13.91	100	10.52

**Figure 11:**
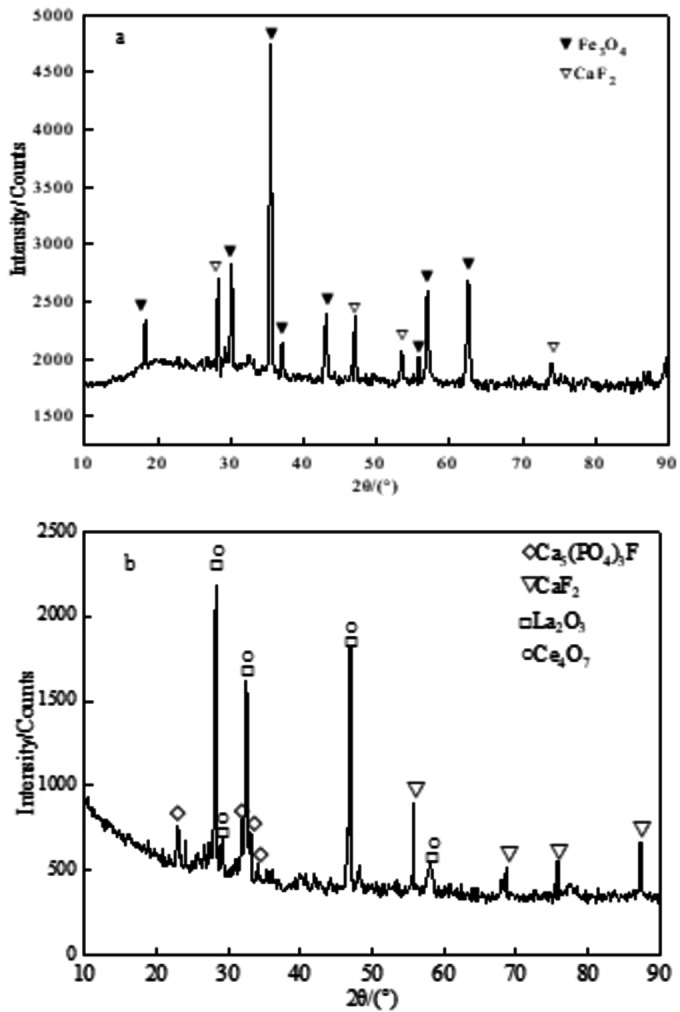
XRD pattern of magnetic the concentrate(a) and tailing(b)

Furthermore, This can be seen from Tables 4 and 5 that the REO and Sc_2_O_3_ grade of magnetic separation tailings is 11.98 % and 0.013 %, respectively. After the subsequent flotation and leaching process, rare earth, Sc_2_O_3_ can be effectively extracted and separated from magnetic separation tailings, which can be used as final products.[^23^, ^24^, ^25^] The XRD patterns of magnetic separation concentrate and tailings are shown in Figures 11(a) and 11(b). The main mineral phases in concentrate are magnetite (Fe_3_O_4_) and (fluorite) CaF_2_, fluorite in concentrate can be removed by grinding and reverse flotation.^[26]^ Meanwhile, the main mineral phases in magnetic separation tailings are REO, CaF_2_, Ca_5_(PO_4_)_3_F, indicating that hematite can effectively reduce strong magnetic Fe_3_O_4_ through the magnetization roasting process. The results also demonstrate that the bastnaesite and monazite can be effectively decomposed into REO and Fe_3_O_4_, and REO can be effectively separated using a low‐intensity magnetic separation technique.

## Conclusions

This article presents a green and efficient process for utilizing LIMS tailings. To this end, MLA analysis was employed to analyze tailings, indicating that LIMS tailings primarily consist of hematite, fluorite, bastnaesite, amphibole, and dolomite. Iron minerals and rare earth minerals are partly in monomer form, and partly in the form of fine particles or inclusions. Then the roasting‐magnetic separation was conducted and the iron grade increased to 65.49 %. Fluorite compounds in magnetic concentrate were removed by grinding and reverse flotation and a high‐grade iron concentrate was achieved. The performed analyses revealed that the magnetization roasting of LIMS tailings reduced hematite to strong magnetic magnetite and decomposed rare earth minerals into REO. Both iron minerals and REO were effectively separated using a low‐intensity magnetic separation process. This article has substantial industrial potential and provides a foundation for the recovery of valuable elements in LIMS tailings.

## Conflict of interest

The authors declare no conflict of interest.

1

## Data Availability

Research data are not shared.
